# American Medical Association, Section on Stomatology

**Published:** 1902-11

**Authors:** 


### AMERICAN MEDICAL ASSOCIATION, SECTION ON STOMATOLOGY.

(Continued from page 772.)

### DISCUSSION ON DR. LATHAM'S PAPER.

(For Dr. Latham's paper, see page 719.)

*Dr. M. L. Rhein,* New York.—I do not think there is any more important subject in our specialty than that of the pulp. It is impossible for me to discuss Dr. Latham's paper as it deserves to be discussed from merely hearing it read yesterday. It is so replete with pertinent inquiry and valuable data that it requires a very careful study of the details.

The large percentage of pathological conditions of the pulp is a point conforming closely to clinical practice in dental work, and is highly important in our consideration of the pulp question. When we consider the nature of the pulp, we appreciate the difficulty in finding a normal, physiological pulp after maturity. With the congestion of the vessels there is a departure from the normal pulp. I have long been under the impression that the average dental practitioner has a very small conception of the enormous percentage of pulp-nodules in various stages of formation in pulps which they remove. These pulp-stones unquestionably go through a series of formation. In our microscopical work we have learned to differentiate the nodules resembling little pointed diamonds and the seed-like formations. The fact brought out by Dr. Latham, that these nodules are found at or near the apex in some cases, and in others towards the coronary portion, is also very interesting from clinical experience. My own theory is that they are largely due to inflammatory action that has proceeded from the exterior through the dentine. I have never seen them unaccompanied by external breaks in the enamel and tooth-structure. On the other hand, I commonly open a tooth upon a diagnosis of pulp-stones and find none until I reach the end of the root, and this in teeth without break on the outer periphery of the enamel. It seems logical that these nodules are due to the lime salts through some irritation in the blood-supply being deposited in the cell tissue. My discussion is not from a microscopical stand-point, but entirely from a clinical.

Dr. Latham spoke of the results of embolism in the arteries as a result of pulp removals and the trouble following such engorgement. The point I want to consider is, what relation has the removal of the pulp upon the circulatory condition of the peridental membrane? From clinical experience I believe that in many cases there has been no break in the enamel structure, but when the pulp-tissue is pathological there has resulted loosening of the teeth, elongation of the incisors, and pyorrhœal symptoms. This condition has been cured by the removal of such pulps. My own view is that the grave pathologic condition of the pulp interfered with the nutrition of the pericemental tissue coming from the dental artery. It does not seem unreasonable that through a perversion of nutrition due to a pathologic condition the pulp is affected and possibly the formation of pulp-stones fostered. Is it natural to say that the pericemental tissue is not deprived thereby of its natural source of nourishment? and when we cut off the entrance of that amount of blood at the end of the root is it natural to suppose that the pericemental tissue should not receive the benefit of that additional nutrition?

I should like to ask Dr. Latham whether the glands she has had under observation show a physiologic or pathologic condition? In the use of the radiograph in dental work I have found in a large number of loosened teeth these glands well defined.

*Dr. R. R. Andrews.*—I am not in a position to discuss Dr. Latham's paper. I should want to read it carefully, and could not do it justice otherwise. I shall therefore speak of the tooth as we see it through the microscope. I believe the odontoblastic layer grows as the tooth grows down. We have a whole mass of hypoblastic and mesoblastic tissue, and the formation takes place nowhere near the hypoblastic tissue. What I fail to see is how the hypoblastic tissue can get in between there in the forming teeth. There is a great deal of discussion about the basement membrane. I do not believe there is any such thing as a basement membrane. We find a membrane in the dentine when it calcifies, and when the enamel is formed we find the same layer. That, to my mind, is the first stage of calcification, and has no appearance to me of being a membrane. I cannot see how it is possible in that tissue to get a hypoblastic tissue. I am not fully up in my reading; most of my work was done ten or fifteen years ago. This work is work for young eyes, and Dr. Latham will have to call a halt pretty soon if she wants to keep her eyes. I think it would be well to prepare a paper upon the points suggested in Dr. Latham's paper, because there is so much contained in it.

In regard to the calcification of the pulp. The pulp is over-irritated in some way from the cemental surface, and a cell within the pulp is stimulated and there is beginning calcification. This calcification irritates some other nerve-tissue and forms osteoblasts. This may be local or general. I have seen pulps in which there are little spicules of bone forming all over it and sometimes on the edge of the nerve through the odontoblastic layer. The fibre-cells which line the pulp, the pear-shaped cells, go only a little distance, but all the other cells take on activity, as in the deeper portions, and new tissues are formed. But, secondary dentine is not normal. The whole pulp in an elderly person may calcify. I believe it will in time be found that the nerve-fibre enters the dental canal.

*Dr. Eugene S. Talbot.*—In my dental histologic work it has been impossible to find many pulps which could be considered normal. Pulp-stones are common in teeth with exposed roots the result of interstitial gingivitis or pyorrhœa. We are now experimenting with the third molar tooth, which we can easily secure and can get them in all stages. The pulps are in a formative stage in most all of these teeth, and therefore I believe that better work will be done on the third molar, except where we extract the tooth for the correction of irregularities.

*Dr. Andrews.*—Dr. Rhein spoke of the immense number of pathologic pulps. We must remember that we see only those which we take out. The largest proportion of pulps are normal. That is a correction which I want to make.

*Dr. G. Lenox Curtis,* New York City.—It has occurred to me that little has been said upon the physical condition of those people from whom the pulps have been secured. And that opens up a subject which should be investigated. My experience is that the patients have histories of long illness, and that their physical condition is far below normal, yet I have felt that this observation was contradicted when I have seen patients who gave a history of perfect health from childhood and found these pulp-stones in apparently perfect teeth. I have observed these pulp-stones in old, diseased, and apparently normal teeth, and have found them of various kinds and sizes; some were just barely visible under powerful magnifying glass, and others filled the entire cavity.

*Dr. A. E. Baldwin,* of Chicago.—In all our investigations we must have a normal stand-point, and I can conceive how two people of like earnestness may arrive at totally different conclusions because of the starting-point of each. The medical student listens to the breathing of a patient and observes the general conditions. He should then be expelled from the sick room, and observe only those said to be in health, so that he may arrive at a perfect knowledge of health before examining disease. We are too apt to start on the observation of disease. We must know first the conditions in health.

*Dr. E. A. Bogue,* New York City.—Dr. Latham spoke against the too frequent extirpation of the pulp, taking the ground that the pulp was not only the formative organ, but that its persistence in life was the health of the tooth. Recently in operating I found a condition of senile decay. Later, asking my son the definition of senile decay, he said he supposed it was a condition existing when the pulp-chamber had been filled or nearly so with secondary dentine, thereby depriving the tooth of its nourishment and of that feature which we call vitality. Hence the tooth breaks down readily. I think the answer bears upon the information given us by Dr. Latham and upon the advice of those gentlemen who advise extirpation at an early stage of the pulp in the apparently normal tooth. The remaining tissues of a tooth which has suffered extirpation of its pulp are placed in the condition of the senile tooth. I think we should emphatically condemn the too frequent extirpation of the pulp.

*Dr. M. L. Rhein.*—What is an early age? and What are normal pulps? are mooted questions, and the most important question is which is the lesser evil of the two. I think all dentists recognize the evil they must do in removing pulps for certain pathologic conditions, but they are committing what they consider the lesser evil. The dentist prefers to do that and preserve the stability of the root of the tooth, for as practical men we know that if we can preserve the integrity of the attachment of the root to the peridental tissues, as far as the crown of the tooth is concerned it matters very little. Dental art has reached such a stage that that part of the tooth can always be made presentable. I only want to present the point, Which is the lesser evil, to lose the tooth entirely at a very early period, or to preserve the root?

*Dr. Andrews.*—In regard to the using up of the odontoblasts, the question has been much discussed of the joining of the cells as the larger portion of the pulp and newly formed tooth closes in. I have seen them joining themselves, or thought they were. There are many points of that kind I would like to hear discussed, but I do not think they had better be taken up at this time.

*Dr. Vida A. Latham* (closing the discussion).—I am not discussing pathology. That could be better taken up twenty years hence. The point is, what is the tooth-structure based on? I make the argument that it is based upon the formative organ, the pulp. I am not discussing the formation of dentine, enamel, or anything else. I want to get back to normal histology, and I do not like such terms as myxomatous tissue. This is but beginning work. My time has been spent in collecting material. I have taken up the theories of the *function* of the pulp, not pathology. The pulp is a more important organ than we will allow. The nervous mechanism of the pulp is not proved. The histological basis is not proved. Professor Paul and myself, I believe, are the only two that have made special efforts to prove that there is more of a basis to the pulp than most investigators have allowed. We have also gone to work to get the nervous mechanism, and I believe that the photograph which I showed demonstrated the vasomotor connection with the pulp. That, I hope, is practically put on a foundation, that is, microscopically, clinically, and photographically. The connection with the odontoblastic layer I am trying to prove, and have succeeded in tracing the nerve-fibres to that point, but have not had time to continue the connection into the cell. The work has been carried on for fifteen years which included the making of thousands of stains, and to-day it is imperfect.

Then the structure of these cells I have not taken up. I have taken up other people's theories and I have argued my paper on these, because to get back to the origin of the odontoblasts we must consider function and physiologic relations.

The connection of the pulp-stones I argued on Dr. Anderson's paper, not my own, and showed the photograph to prove her paper. I mentioned that in my sections these calcareous bodies were formed all the way through. The origin of these is another thing. If they are found, how can they be formed from the odontoblasts? I rather think they cannot be, unless we have the odontoblasts in this connective tissue working in a way we do not realize. I want my knowledge to be based on histology. I do not want the dental tissue with no other analogy in the human body, hence I have been working upon the origin of the odontoblast, the mesoblasts, and the function of the secretory organs, but I have not proved all the points. Further, I would say that a good deal of argument has been based upon pathologic conditions. This paper is simply to show you that when a man speaks of a layer of cells forming he speaks of hundreds of layers. The point of my paper is to prove the nervous relations, the structural basis of the pulp, the varieties of the odontoblasts, which is the true odontoblast, which the formative cell, and its vasomotor connection with the tooth, and then later take up the pathologic function. This work should be illustrated by carefully prepared photographs, and I hope to get something that I shall call normal to make a basis for the work.

DISCUSSION ON DR. TALBOT'S PAPER.

(For Dr. Talbot's paper, see page 701.)

*Dr. Vida A. Latham.*—I want to emphasize the value of comparative histology and anatomy. Dr. Talbot's paper has pointed out to me that there is some ground for my statements regarding the hypoblastic layer. He is considering it in the evolution of the pulp. He gives as his analogue the kidney, which organ until Heidenhain was little understood, but to-day is placed as a purely secretory and excretory organ. I think we will make a beautiful analogy between the protoplasm of the kidney and the odontoblastic layer.

Regarding the formation of enamel, the question comes up, Does enamel form after a tooth is practically made, or do we have a continued formation of enamel? I believe we do. The use of the dental irregularities apparatus shows this. It makes a dent or impression and by friction a small pin-hole, and you will find a projection of enamel. I believe that enamel does not always stop forming when it covers the tooth, any more than skin ceases to grow. In the rat the blood reaches the enamel. This is another point to prove the value of comparative histology.

*Dr. Talbot* (closing the discussion).—I have had this paper on my mind for a number of years, and I was led to read it at this meeting because a gentleman, in reviewing one of my books a short time ago, took exception to the question and answer, "Is degeneracy or arrest of development of jaws and teeth a cause of decay? Answer: Yes, it is the principal cause." Then I went on to say that it was a matter of record that teeth decayed more rapidly on the upper jaw than on the lower. The reviewer of my book said that that was not true. He stated also that the farther away from the salivary ducts the greater the decay of the tooth, therefore teeth decay more rapidly on the lower jaw than on the upper. It seems to me that that is rather a narrow way of looking at the subject, and is ignoring the constitutional cause producing decay. We must take up the evolution of the pulp, as Dr. Latham has said, as the principal argument. It is the pulp itself that we have come to study from its evolution to its histology in order to understand decay of the teeth. In taking a broad view of the decay of teeth and diseases of the alveolar process, it stands to reason, from the paper I have read this morning, that teeth are degenerated from the lower animals, in which series of animals we have teeth with open pulps, and these pulps having large cavities, the blood flowing to them keep them in a normal, healthy condition. As we ascend into the cycle of man the ends of the roots close up and they become to a certain extent foreign bodies; and here comes the point in regard to senile degeneration. The point is illustrated by a man whom I have in mind. This man is the editor of a journal, but on account of a change in the Administration he is appointed postmaster and has a double amount of work to do. He had been a patient of mine for over thirty years, and had until recently as hard teeth as ever before. They were like flint and without decay. In about six months after becoming postmaster, with the large amount of work upon his hands, he had neurasthenia, and as a result his teeth cut like horn and had a general pathologic appearance. This senile condition comes on in healthy people after forty years of age, and when in addition the nervous system is wrought up not only do the teeth decay more rapidly, but the other structures of the body give out as well.

The rudimentary enamel-organs we find in all the structures of the human as well as in the animal. There is no question in my mind but that Magitot and other investigators brought out the true conditions of these cells which Black calls glands, nothing more than rudimentary organs which would have gone to form extra teeth, as we find in some animals forty-two and seventy-two. These are the rudimentary organs which are not used at the present time on account of the law of economy of growth. In order to understand the histology and pathology of the teeth, we must study the evolution of the pulp. I do not see why that subject has not been taken up in our schools.

Adjourned to two P.M.

(To be continued.)

